# A detached petal disc assay and virus-induced gene silencing facilitate the study of *Botrytis cinerea* resistance in rose flowers

**DOI:** 10.1038/s41438-019-0219-2

**Published:** 2019-12-01

**Authors:** Xiaoqian Cao, Huijun Yan, Xintong Liu, Dandan Li, Mengjie Sui, Jie Wu, Hongqiang Yu, Zhao Zhang

**Affiliations:** 1 Beijing Key Laboratory of Development and Quality Control of Ornamental Crops, Department of Ornamental Horticulture, College of Horticulture, China Agricultural University, Beijing, 100193 China; 2 Flower Research Institute of Yunnan Academy of Agricultural Sciences, Kunming, 650205 Yunnan China

**Keywords:** Biotic, Plant breeding

## Abstract

Fresh-cut roses (*Rosa hybrida*) are one of the most important ornamental crops worldwide, with annual trade in the billions of dollars. Gray mold disease caused by the pathogen *Botrytis cinerea* is the most serious fungal threat to cut roses, causing extensive postharvest losses. In this study, we optimized a detached petal disc assay (DPDA) for artificial *B. cinerea* inoculation and quantification of disease symptoms in rose petals. Furthermore, as the identification of rose genes involved in *B. cinerea* resistance could provide useful genetic and genomic resources, we devised a virus-induced gene silencing (VIGS) procedure for the functional analysis of *B. cinerea* resistance genes in rose petals. We used *RhPR10.1* as a reporter of silencing efficiency and found that the rose cultivar ‘Samantha’ showed the greatest decrease in *RhPR10.1* expression among the cultivars tested. To determine whether jasmonic acid and ethylene are required for *B. cinerea* resistance in rose petals, we used VIGS to silence the expression of *RhLOX5* and *RhEIN3* (encoding a jasmonic acid biosynthesis pathway protein and an ethylene regulatory protein, respectively) and found that petal susceptibility to *B. cinerea* was affected. Finally, a VIGS screen of *B. cinerea*-induced rose transcription factors demonstrated the potential benefits of this method for the high-throughput identification of gene function in *B. cinerea* resistance. Collectively, our data show that the combination of the DPDA and VIGS is a reliable and high-throughput method for studying *B. cinerea* resistance in rose.

## Introduction

Fresh cut roses are among the most popular ornamental plants, generating billions of dollars in trade annually and accounting for more than one-third of the total cut flower industry worldwide^1^. The supply of cut roses is heavily reliant on the logistics of long-distance transportation, as cut roses are produced mainly in developing countries with low labor costs and suitable climates, such as Kenya, Colombia, Ecuador, and China, whereas the consumer market is mainly in the developed countries of North America, Europe, and Japan. Globally, the average transport distance for each rose, from farm to customer, is more than 1500 km^2^. During this long-distance transportation, cut roses are challenged by various biotic and abiotic stresses, among which gray mold disease caused by *Botrytis cinerea* causes the most severe postharvest losses.

*B. cinerea* is among the world’s most notorious plant pathogens, causing gray mold disease in over 200 dicotyledonous and monocotyledonous species^3^. Germinated *B. cinerea* conidia produce secondary metabolites and phytotoxic proteins that induce host cell death during the penetration of the host epidermis^4^. In rose, *B. cinerea* infection leads to necrotic lesions on petals, and symptoms develop rapidly during postharvest transport, during which flowers are packed in boxes with high relative humidity^5^.

Despite the economic importance of this pathogen in roses, research on the rose–*B. cinerea* interaction has been limited compared to research on the pathogen’s behavior in other plants, such as the model plant *Arabidopsis thaliana* (Arabidopsis) and the Solanaceous species *Nicotiana benthamiana* and tomato (*Solanum lycopersicum*), which are also susceptible to *B. cinerea*. However, research on *B. cinerea* infection in Arabidopsis and *N. benthamiana* has focused on the infection of leaves and, in tomato, that of fruits. In ornamental crops such as gerbera^6^ and rose^4,7^ by contrast, *B. cinerea* mainly infects flower petals, damaging the most economically important organ of these plants, while the leaves, fruits, stems, and sepals are rarely infected or of little importance. Petal cells begin to senesce immediately after harvest^8^, which facilitates infection by necrotrophic fungi such as *B. cinerea*. In addition, petal epidermal cells are a unique plant tissue with specialized conical structures. The valleys between conical cells may facilitate the survival of airborne *B. cinerea* conidia.

Artificial inoculation is a critical technique in disease phenotyping and thus in studies of the interaction between *B. cinerea* and rose, both for fundamental research and for breeding purposes. Previously, rose was inoculated with *B. cinerea* via the inoculation of whole flowers with fungal conidia; disease severity was scored according to a “disease index” based on a scale of 0–5 (or more), ranging from no infection to the fungus covering the whole flower^9,10^. However, this conventional method cannot be used to accurately quantify disease resistance. We therefore aimed to develop an improved method for artificial inoculation and disease quantification in rose petals. To this end, we designed and optimized a detached petal disc assay (DPDA) for artificial inoculation and accurate symptom quantification of *B. cinerea* infection in rose.

Furthermore, as the functional characterization of rose genes thought to be involved in *B. cinerea* resistance is limited by the low efficiency and long timeframe of genetic transformation (2 years from transformation to flower production), we used an alternative molecular approach involving virus-induced gene silencing (VIGS). This method, in which target genes are knocked down based on double-stranded RNA-triggered RNA degradation, has been widely exploited for gene functional analysis^11^. In VIGS, when a recombinant virus carrying the sequence of a host gene\spreads throughout the plant, the host target gene transcripts are degraded together with the viral transcripts, and the gene of interest in the host plant is therefore silenced. Previously, a VIGS approach using recombinant tobacco rattle virus (TRV) was established for the silencing of genes in rose flowers^12^. Once young plantlets or sprouts are vacuum infiltrated with *Agrobacterium tumefaciens* (Agrobacterium) carrying recombinant TRV-derived vectors, the infected plantlets/sprouts are grown in soil or grafted to a root stock until flowering (which takes approximately 5–15 weeks), at which point it is possible to evaluate their phenotypes, such as flower color, scent, and floral development^12^. Although it has not been reported previously, it is likely that this VIGS protocol could be used to study rose resistance to pathogens. However, the full exploitation of VIGS assays for investigating *B. cinerea* resistance in rose petals could be hampered by the time (5–15 weeks) and labor costs of VIGS. In addition, a large volume of the *Agrobacterium* suspension is needed for the immersion and vacuum infiltration of plantlets, and a large area in a greenhouse or climate-controlled chamber is required to support the growth of rose plants from agroinfiltration until flowering. These limitations have prevented the rapid and high-throughput screening of *B. cinerea* resistance genes in rose flowers.

To address these challenges, we developed a VIGS method for silencing candidate genes in detached rose petal discs, which allows the rapid identification of their function. We demonstrate that this VIGS method, combined with the DPDA, can be used for high-throughput screening of candidate *B. cinerea* resistance genes.

## Results

### Inoculation of detached rose petal discs with *B. cinerea*

To establish the DPDA, we obtained detached petals from the outermost whorl of rose (*Rosa hybrida*) flowers at stage 2 of flower opening^13^ and punched out 15-mm discs from the center of the petals. We placed the petal discs on a wet filter paper in a 9-cm petri dish and inoculated the abaxial or adaxial surface of the discs with 2 μL of *B. cinerea* conidia at 1 × 10^4^/mL. However, we observed only tiny disease spots up to 72 h postinoculation (hpi). This may have been due to the difficulty of controlling the wetness of the filter paper under this method, which resulted in insufficient humidity for *B. cinerea* infection (Supplemental Fig. S1a).

**Fig. 1:**
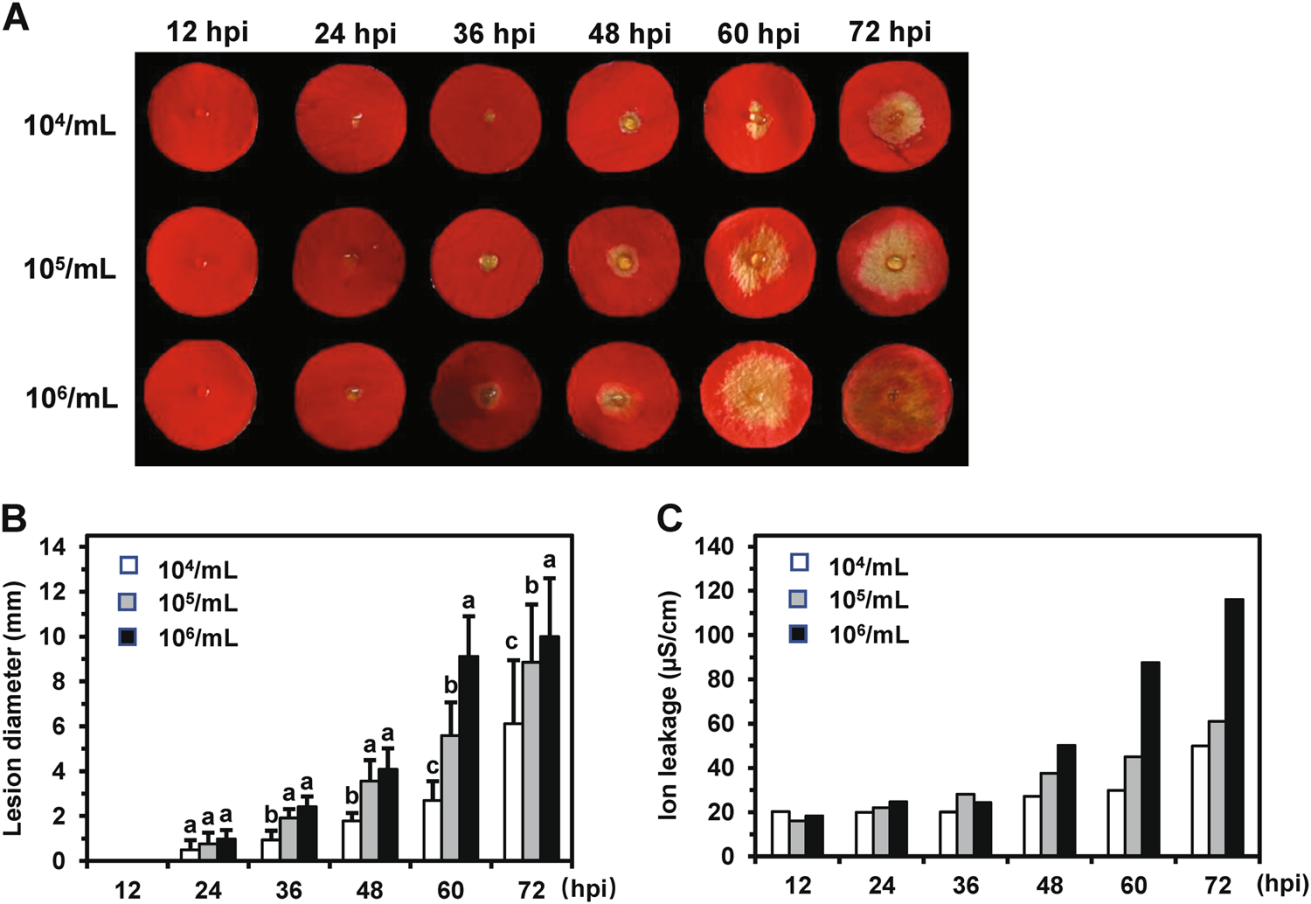
Optimization of *Botrytis cinerea* inoculation on rose petal discs. **a** Petal discs were inoculated with different concentrations of *B. cinerea* conidia. Disease lesions were observed at 12, 24, 36, 48, 60, and 72 hpi. **b** Statistical analysis of disease lesions following inoculation with different concentrations of *B. cinerea* conidia. The graph shows the average lesion size from three biological replicates (*n* = 48) with the standard deviation. Significant differences are indicated by lowercase letters according to Duncan’s multiple range test (*P* < 0.05). **c** Quantification of cell death triggered by *B. cinerea* infection of rose petal discs. The graph shows ion leakage values measured at 12, 24, 36, 48, 60, and 72 hpi. The ion leakage changes resulting from petal cell death were consistent with the disease lesion diameter. This experiment was repeated two times with similar results.

To overcome this problem, we placed petal discs on 0.4% (w/v) agar and inoculated the discs using the same method. Through this approach, we observed clear, extended, and consistent lesions on all inoculated discs on either the adaxial or abaxial side at 48 hpi (Fig. 1a; Supplemental Fig. S1b). We subsequently inoculated the adaxial side of discs on 0.4% (w/v) agar.

To determine the optimal concentration of fungal spores for inoculation as well as the optimal time point for phenotyping, we inoculated petal discs with 1 × 10^4^/mL, 1 × 10^5^/mL, and 1 × 10^6^/mL conidia and measured disease lesions at 12, 24, 36, 48, 60, and 72 hpi. At 60 and 72 hpi, we observed statistically significant differences in the disease symptoms (lesion diameter) of discs inoculated with the three different conidia concentrations (Fig. 1b). In addition, we observed ion leakage resulting from petal cell death, consistent with the diameters of the lesions (Fig. 1c). This further suggested that although punching out a disc causes mechanical damage and, hence, some ion leakage, the cell death induced by *B. cinerea* further affects the ion leakage of petals. For the DPDA, we ultimately decided to use 2 μL of conidia at 1 × 10^5^/mL delivered to the adaxial side of the petal discs and to measure and statistically analyze disease lesions at 60 hpi.

### Characterization of the disease susceptibility of 22 rose genotypes

**Fig. 2:**
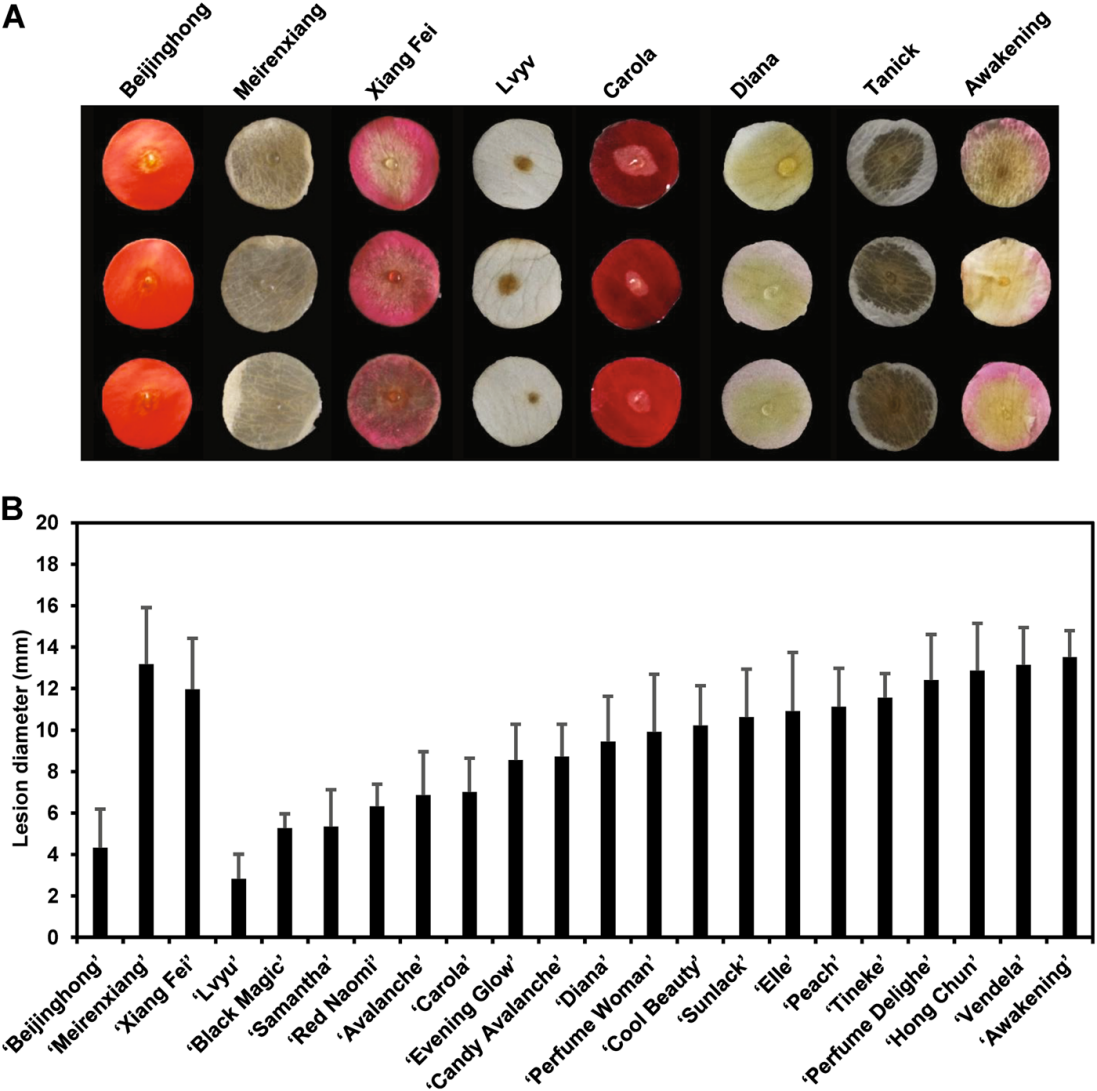
Quantification of *B. cinerea* resistance in various rose cultivars determined using the detached petal disc assay. **a** Sample images of petal discs from each cultivar after infection with *B. cinerea* (photographed at 60 hpi). **b** Average lesion size measured at 60 hpi from at least three biological replicates of each cultivar (*N* ≥ 48 for each cultivar) with the standard deviation.

Using the DPDA, we investigated disease susceptibility in a collection of rose cultivars. To this end, we used three cultivars bred by our lab as controls: ‘Beijinghong’, which is relatively resistant to *B. cinerea*, and ‘Meirenxiang’ and ‘Xiang Fei’, which are susceptible cultivars based on our observations in the field. We showed that the lesions of ‘Beijinghong’ exhibited an average diameter of less than 5 mm at 60 hpi, indicative of significantly more resistance than in the two susceptible cultivars (both of which exhibited lesions with average diameters over 12 mm; Fig. 2). The results of the DPDA for ‘Beijinghong’, ‘Xiangfei’, and ‘Meirenxiang’ were consistent with their susceptibility observed in the field, suggesting that the lesion diameter observed by the DPDA is a good representation of flower fungal resistance.

We further tested a collection of 19 rose cultivars and found substantial differences in their susceptibility to *B. cinerea*. The cultivar ‘Lvyu’ was the most resistant, with an average lesion size at 60 hpi of less than 3 mm; some cultivars, such as ‘Black Magic’, ‘Samantha’, ‘Red Naomi’, ‘Avalanche’, and ‘Carola’, were moderately susceptible, with an average lesion size of 5–8 mm; and others, including ‘Perfume Delight’, ‘Hong Chun’, and ‘Vendela’, were highly susceptible to *B. cinerea*, with lesion sizes of 12–15 mm (Fig. 2).

### TRV-based VIGS in detached rose petals

It was previously demonstrated that TRV-mediated VIGS can be used in rose as a tool for the functional analysis of genes involved in flower development and the determination of petal color, and fragrance^12^. To assess the roles of candidate genes in *B. cinerea* resistance signaling, we aimed to optimize TRV VIGS by identifying rose cultivars that would be suitable for inoculation and efficient VIGS in petal discs. First, an appropriate cultivar should exhibit clear lesions after inoculation, as the edges of the lesions are not very clear in some cultivars, hampering their measurement (e.g., ‘Marie-Victorin’, Supplemental Fig. S2). Second, an ideal cultivar should be moderately resistant to *B. cinerea*, making it suitable for the screening of both positive regulators (whose knockdown through gene silencing results in larger disease lesions) and negative regulators (whose knockdown results in smaller lesions) of defense signaling. Third, dark flowers are more suitable for the analysis than pale flowers because the fading of dark-colored petals caused by the silencing of anthocyanin-related genes can be used as a reporter of silencing efficiency (similar to the *Phytoene Desaturase* (*PDS*) gene, used as a reporter of silencing efficiency in plant leaves)^14^.

We therefore selected four rose varieties with clear lesions, moderate resistance, and dark color—‘Samantha’, ‘Carola’, ‘Black Magic’, and ‘Red Naomi’—as candidates for VIGS assays. Different cultivars may show different efficiencies of TRV-mediated gene silencing^14,15^. To test the efficiency of gene silencing in the four candidate cultivars, we used the *RhDFR1* (*Dihydroflavonol 4-reductase 1*) gene as a reporter. *RhDFR1* is required for the accumulation of anthocyanin in rose flowers, and its silencing has been shown to lead to bleached flower color symptoms throughout rose flowers^12^. To determine whether the silencing of *RhDFR1* induced similar bleaching in rose petal discs, we vacuum infiltrated a 1:1 mixture of *Agrobacterium* carrying *pTRV1* and *pTRV2::RhDFR1* into rose petal discs. However, we did not observe increased color fading in the petals up to 10 days postinfiltration (Supplementary Fig. S3). It could be that the pigments have already accumulated in the petals of fully developed flowers and that the silencing of *RhDFR1* does not reduce the level of pigments already present in the petals. Thus, we concluded that, unlike the situation in complete flowers (Yan et al.^12^), *RhDFR1* cannot be used as a silencing reporter gene for VIGS in petal discs.

**Fig. 3:**
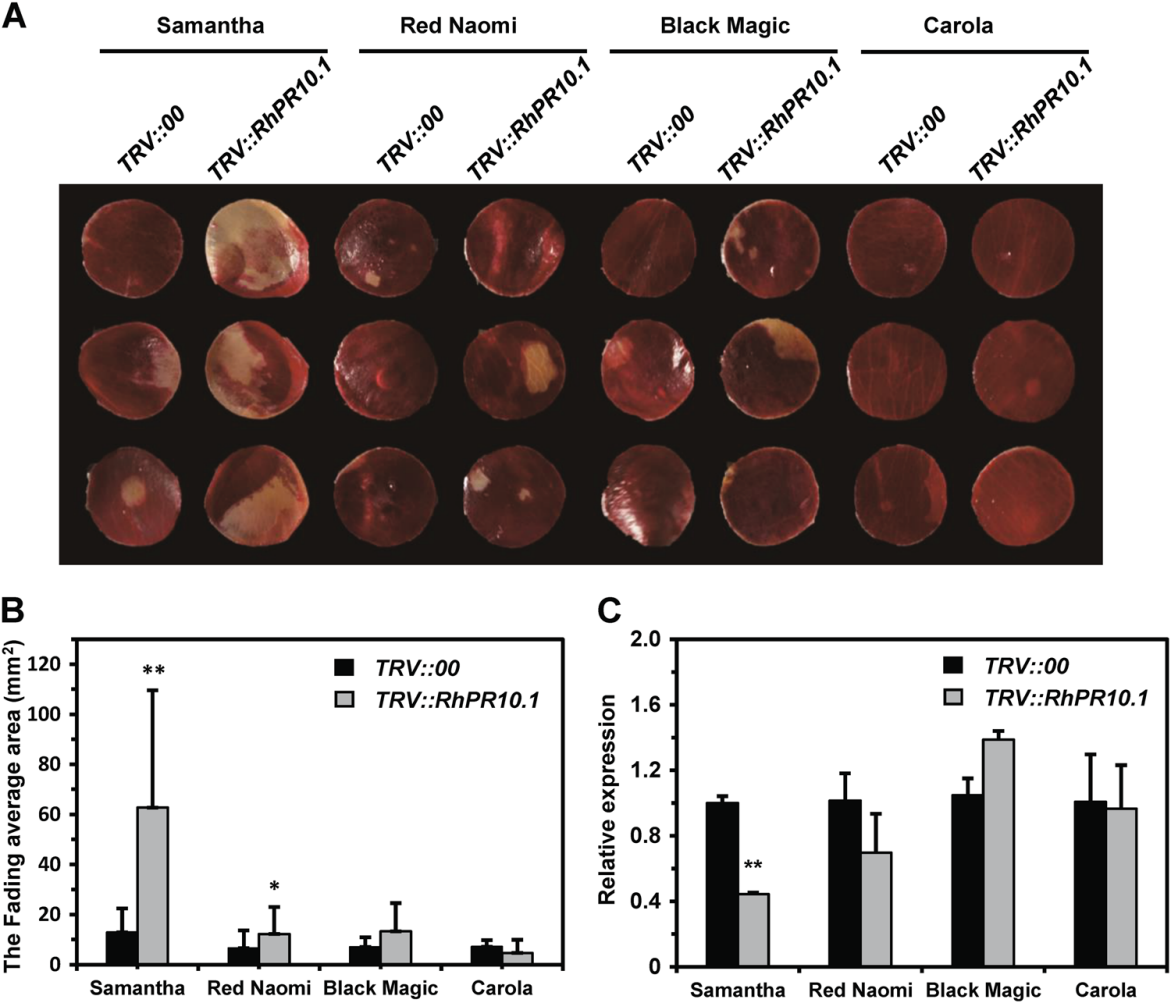
Comparison of silencing efficiency in the cultivars ‘Samantha’, ‘Red Naomi’, ‘Black Magic’, and ‘Carola’. **a** The strongest color fading upon *RhPR10.1* silencing was observed in ‘Samantha’; all photographs were taken at 14 days postinfiltration. **b** Total area of color fading in the four cultivars. The average size of the color-fading area from three biological replicates of each cultivar (*N* = 48) with the standard deviation is shown. **c** Relative expression of *RhPR10.1* at 14 days postsilencing. *RhACT5* was used as an internal housekeeping gene control. Statistical analysis was performed using Student’s *t* test; *, *P* < 0.05; **, *P* < 0.01.

Recently, a suppressor of petal senescence, RhPR10.1, was identified^16^. Knockdown of *RhPR10.1* expression accelerates senescence in rose petals, resulting in a color-fading phenotype. To compare the silencing efficiency of this gene in the four candidate cultivars, we vacuum infiltrated *Agrobacterium* bacteria containing the recombinant TRV vectors into the petal discs of the four cultivars to silence their *RhPR10.1* genes. At 2 weeks postinfiltration, *RhPR10.1*-silenced ‘Samantha’ and ‘Red Naomi’ petal discs showed significantly increased fading compared with control petals, while the other two cultivars showed no increase in fading (Fig. 3a, b). We further used qPCR to detect the silencing efficiency in the four cultivars. We found that ‘Samantha’ showed the most significant decrease in *RhPR10.1* expression upon infiltration with *TRV::RhPR10.1* (Fig. 3c). These results were consistent with the observation that fading was most pronounced in the petal discs of ‘Samantha’ and suggested that this was the most suitable cultivar for our purposes, as it was appropriate for the DPDA because of its moderate resistance and for VIGS because of its high silencing efficiency.

To determine whether the agroinfiltration of recombinant TRV vectors affected the responses of rose to *B. cinerea*, petal discs were vacuum infiltrated with water, infiltration buffer, or *Agrobacterium* carrying recombinant TRV vectors. The infiltrated petal discs were subsequently inoculated with conidia at 1 × 10^4^/mL, 1 × 10^5^ /mL, or 1 × 10^6^/mL. However, we did not observe a significant difference in the disease lesions of petal discs infiltrated with water, infiltration buffer, or *Agrobacterium* carrying recombinant TRV at 60 hpi (Supplemental Fig. S4).

### JA plays a role in regulating *B. cinerea* resistance in rose petals

Previous studies have shown that in the model plant Arabidopsis, the phytohormone jasmonic acid (JA) plays key roles in resistance to necrotrophic pathogens (e.g., *B. cinerea*) and the response to mechanical damage, while salicylic acid (SA) often acts antagonistically to JA^17^.

**Fig. 4:**
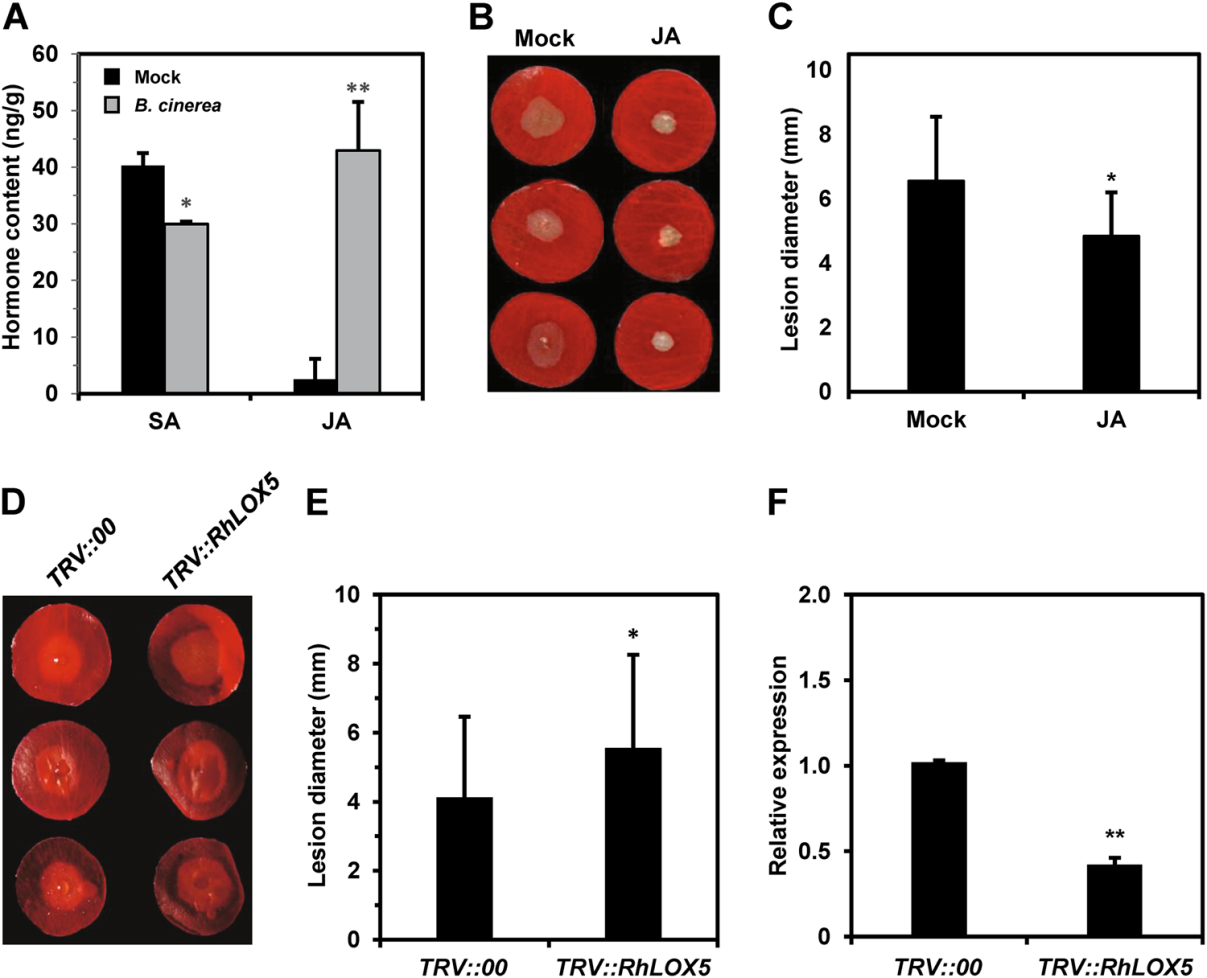
Jasmonic acid regulates *B. cinerea* resistance in rose petals. **a** At 48 hpi with *B. cinerea*, the content of the endogenous hormone JA was significantly increased in petal discs, while that of SA was decreased. **b** Exogenous JA treatment significantly enhanced *B. cinerea* resistance in rose petal discs. **c** Quantification of disease lesions on JA-treated and mock-treated rose petal discs. The graph shows the average diameter of disease lesions at 60 hpi; data are from at least three biological replicates of each cultivar (*N* ≥ 48) with the standard deviation. **d** Compromised *B. cinerea* resistance upon silencing of *RhLOX5*, which encodes a key enzyme of the JA biosynthesis pathway. (**e**) Quantification of *B. cinerea* disease lesions on *TRV::RhLOX5*- and *TRV::00*-inoculated rose petal discs. The graph shows the average diameter of the disease lesions at 60 hpi; data are from at least three biological replicates of each cultivar (*N* ≥ 48). (**f**) Relative expression of *RhLOX5* at 6 days postsilencing. All statistical analyses were performed using Student’s *t*-test; *, *P* < 0.05; **, *P* < 0.01; hpi hours postinoculation.

In our DPDA system, discs are punched from rose petals. To determine if the mechanical damage caused by this process influences *B. cinerea*-mediated changes in endogenous JA levels, we assessed the changes in endogenous JA and SA levels in petal discs during *B. cinerea* infection. We used the DPDA to determine endogenous JA and SA levels by high-performance liquid chromatography (HPLC) at 48 hpi. We found that petal discs infected with *B. cinerea* exhibited a JA content that was almost 10 times higher than that of mock-inoculated discs, whereas the SA content was significantly reduced (Fig. 4a). These results showed that although the DPDA causes mechanical damage to petals, *B. cinerea* infection induces significant changes in phytohormone content in petal discs. The petal discs are therefore representative of the entire plant in these experiments. These results further suggest that JA plays an important role in defense responses in rose petals, as in Arabidopsis.

To test this possibility, we applied 50 μM exogenous JA to rose petal discs and inoculated them with *B. cinerea* 24 h later. The diameter of the disease lesions at 60 hpi was significantly decreased in these petal discs compared with control discs that were not treated with JA (Fig. 4b, c). This experiment confirmed that JA plays a key role in rose defense against *B. cinerea*.

To confirm that VIGS can be used to study *B. cinerea* resistance in rose flowers, we silenced a rose lipoxygenase gene, *RhLOX5*, that encodes a key enzyme of the JA biosynthesis pathway. At one week post-recombinant TRV infection, we inoculated *RhLOX5*-silenced and control petal discs with *B. cinerea*. Sixty hours after *B. cinerea* inoculation, we determined the level of disease resistance by evaluating lesion diameter. As expected, the silencing of *RhLOX5* resulted in significantly larger lesions compared with the control petal discs, indicative of compromised *B. cinerea* resistance (Fig. 4d, e). We confirmed the silencing efficiency of VIGS by qPCR (Fig. 4f). These results suggested that JA is required for rose resistance against *B. cinerea* and, importantly, that VIGS in rose petal discs can be used to study genes involved in rose resistance to *B. cinerea*. The DPDA combined with VIGS is a rapid and reliable tool for the functional analysis of rose genes involved in pathogen resistance.

The SA content decreased significantly in the petals at 48 hpi with *B. cinerea*. To examine the role of SA in *B. cinerea* resistance, we applied 50 μM or 100 μM exogenous SA to rose petal discs and then inoculated the discs with *B. cinerea* 24 h later. However, we found no significant difference between the SA-treated petal discs and control petal discs that were not treated with SA (Supplemental Fig. S5).

### The gaseous hormone ethylene is required for *B. cinerea* resistance in rose petals

The gaseous hormone ethylene (ET) is an important signaling component in plant defense against necrotrophic fungi, such as *B. cinerea*^17^. In Arabidopsis, *B. cinerea* infection results in enhanced ET biosynthesis, and ET is considered to be a positive regulator of *B. cinerea* resistance. However, it has been recently suggested that ET may be involved in both negative and positive effects on the plant’s response to *B. cinerea*, depending on the plant species^18^.

**Fig. 5:**
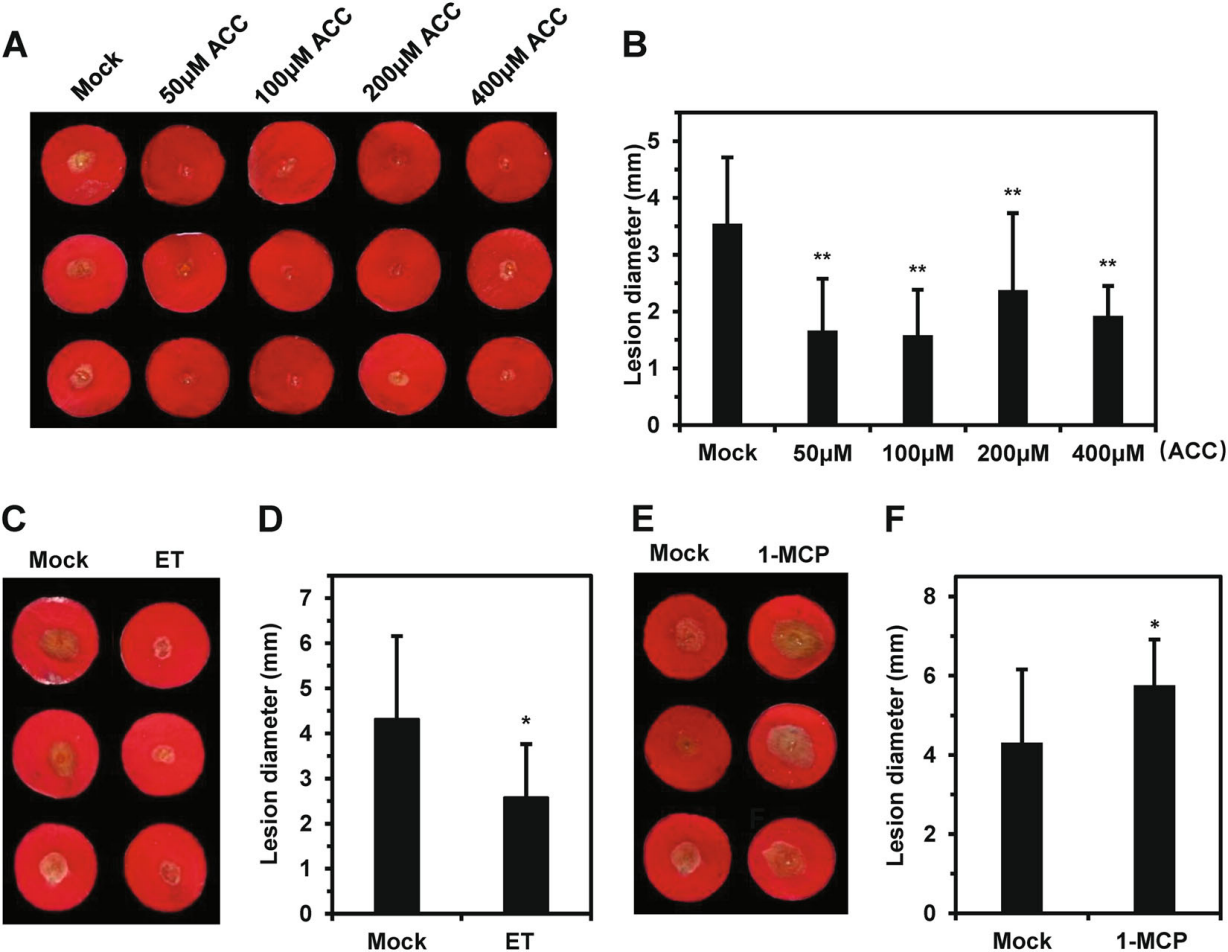
Ethylene is a key regulator of *B. cinerea* resistance in rose petals. **a** Exogenous application of ACC, the direct synthetic precursor of ethylene, enhanced *B. cinerea* resistance in rose petals. **b** Quantification of disease lesion size on rose petals treated with ACC or mock-treated rose petals. The graph shows the average diameter of the disease lesions at 60 hpi. **c** Application of exogenous ethylene (ET) resulted in enhanced *B. cinerea* resistance in rose petals. **d** Quantification of disease lesion size in rose petal discs treated with ET or mock-treated rose petals. The average diameter of disease lesions at 60 hpi is shown. **e** Exogenous application of the ET suppressor 1-MCP compromised *B. cinerea* resistance in rose petals. **f** Quantification of disease lesion size on rose petals treated with 1-MCP or mock-treated rose petals. The average diameter of disease lesions at 60 hpi is shown. All data are from at least three biological replicates (*N* ≥ 48) with the standard deviation. All statistical analyses were performed using Student’s *t* test; *, *P* < 0.05; **, *P* < 0.01.

To clarify the role of ET in *B. cinerea* resistance in rose, we applied various concentrations (from 50 to 400 μM) of 1-aminocyclopropane-1-carboxylic acid (ACC), the direct synthetic precursor of ET, to rose petal discs and then inoculated the discs with *B. cinerea* 24 h later. All four concentrations of ACC used increased rose resistance to *B. cinerea* (Fig. 5a, b). These results suggested that ET plays a positive role in regulating resistance to *B. cinerea* in rose petals. We further treated petal discs with ET gas or 1-methylcyclopropene (1-MCP, an inhibitor of ET that has been used commercially as a postharvest preservative to maintain cut flower freshness) and inoculated the discs at 24 h posttreatment. As expected, ET-treated petal discs showed significantly increased resistance, while 1-MCP-treated petal discs showed compromised resistance, confirming the involvement of ET in *B. cinerea* resistance signaling in rose (Fig. 5c–f). Our results suggested that 1-MCP treatment may have a negative effect on rose resistance to *B. cinerea*.

**Fig. 6:**
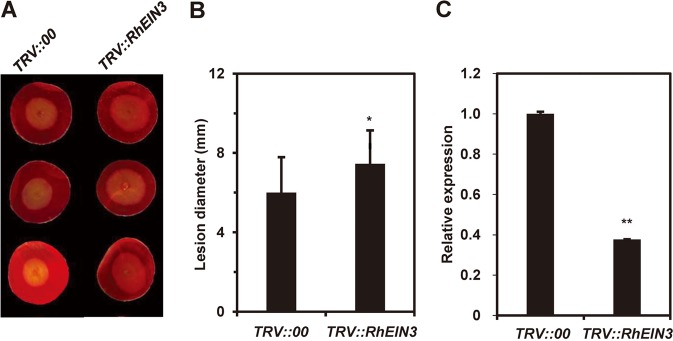
*B. cinerea* inoculation of rose petal discs after virus-induced gene silencing of *RhEIN3*. **a** Petal discs from the rose cultivar ‘Samantha’ were inoculated with empty tobacco rattle virus (TRV) as a control (*TRV::00*) or with a recombinant TRV targeting *RhEIN3* (*TRV::RhEIN3*). Compromised resistance to *B. cinerea* was observed in *RhEIN3-*silenced plants at 60 hpi. **b** Compromised *B. cinerea* resistance upon silencing of *RhEIN3*, which encodes an ethylene regulatory protein. (**c**) Quantification of *RhEIN3* expression in *TRV::RhEIN3*-inoculated petal discs relative to that in the control. Statistical analysis was performed using Student’s *t*-test; *, *P* < 0.05; **, *P* < 0.01.

Next, we used VIGS to silence an ET-related gene in rose petals to further demonstrate that the ET signaling pathway plays an important role in the interaction of rose with *B. cinerea*. In Arabidopsis, the nuclear transcription factor *EIN3* is a critical regulator of ET, and the ethylene-insensitive mutant *ein3* shows increased susceptibility to *B. cinerea*^19^. To silence *EIN3* expression in rose, we inoculated the petal discs with a recombinant virus targeting *RhEIN3* expression (*pTRV::RhEIN3*) or with the empty TRV vector as a control (*pTRV::*00). As expected, *RhEIN3*-silenced plants showed significantly compromised resistance (Fig. 6), confirming the positive involvement of ET in *B. cinerea* resistance in rose. This further suggested that the DPDA and VIGS can be used for studying ET-related defense genes in rose petals.

### Functional analysis of *B. cinerea*-induced transcription factor genes of rose by VIGS and the DPDA

**Fig. 7:**
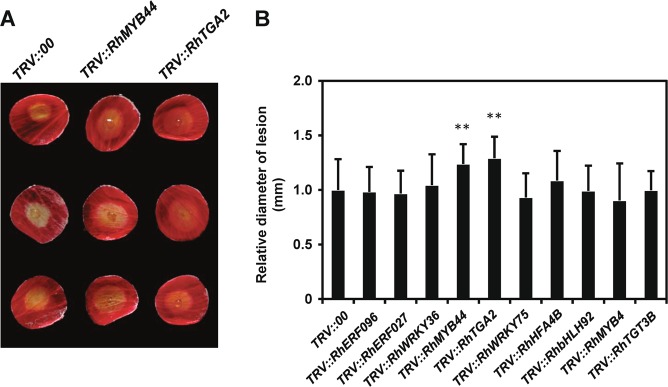
Functional screen of *B. cinerea*-induced rose transcription factor genes by VIGS and the DPDA. **a** Compromised *B. cinerea* resistance symptoms on rose petal discs upon infection with a recombinant tobacco rattle virus (TRV) targeting *RhMYB44* and *RhTGA2*, shown at 60 hpi. **b** Quantification of disease lesions on *BIT*-silenced plants. The graph shows the average diameter of disease lesions with the standard deviation at 60 hpi relative to the control (*TRV::00*). Statistical analysis was performed using Student’s *t* test; *, *P* < 0.05; **, *P* < 0.01.

Previously, 188 *B. cinerea-induced transcription factor* (*BIT*) genes were identified via comparative RNA-seq analysis of rose flowers infected with *B. cinerea*^2^. To illustrate the potential application of our methods to a high-throughput reverse screen of gene function in rose, we performed VIGS to knock down the expression of 10 randomly selected *BIT* genes. To this end, we cloned fragments of selected *BIT* genes varying in length from 200 to 400 bp into the TRV vector and inoculated rose petal discs with these fragments via vacuum infiltration. To test the involvement of the selected *BIT* genes in *B. cinerea* resistance, we subsequently challenged the *TRV::BIT*-inoculated rose petal discs with *B. cinerea*. The *TRV::RhMYB44*- and *TRV::RhTGA2*-inoculated plants showed severely compromised resistance, as evidenced by significantly increased lesion size (Fig. 7). These results indicated that *RhMYB44* and *RhTGA2* are essential for *B. cinerea* resistance in rose. Furthermore, VIGS in combination with the DPDA provided a rapid and high-throughput method for assessing the functions of rose genes in disease resistance in petals.

## Discussion

### The DPDA is a versatile tool for comparative analyses of *B. cinerea* resistance in rose

In the present study, we developed a DPDA that enabled us to quantitatively evaluate *B. cinerea* resistance in rose flowers. Importantly, the results of the DPDA in resistant and susceptible cultivars matched their resistance abilities observed in the field, suggesting that the DPDA is a reliable method for quantifying resistance against *B. cinerea* in rose flowers.

Compared with whole-flower inoculation, our DPDA has clear advantages. First, petal discs are easy to inoculate, as they are relatively flat, whereas rose petals on intact flowers are cambered, so that inoculum drops tend to roll down the flower rather than remain on the petal. Second, the petal discs used in the DPDA require much less space in the climate chamber than do cut flowers in vases. Finally, that detached petal discs could be left on agar plates for over 10 days without dehydration, while the vase life of cut roses is significantly shorter, with cut flowers wilting within a week. This is important because, although *B. cinerea* lesions develop within 3 days, pretreatment with phytohormones or VIGS takes additional time.

Originally, we also considered the use of detached intact petals for the inoculation assay; however, similar to intact flowers, the petals are cambered and, thus, more difficult to inoculate. In addition, intact petals wilt more easily than discs on 0.4% agar plates. Finally, using the entire petal takes up more space in the climate chamber, as only one petal can be placed in a 9-cm petri dish, whereas 16 discs (i.e., 16 independent inoculation repeats) can fit in a petri dish of this size.

A potential disadvantage of the DPDA is that the mechanical damage caused by punching induces the accumulation of JA, which is also a resistance signal against necrotrophic fungal pathogens, such as *B. cinerea*. However, we circumvented this concern by including proper controls. For example, we found that petal discs inoculated with *B. cinerea* accumulated significantly higher levels of JA (almost 10 times as high; Fig. 4a) than mock-inoculated discs prepared in the same fashion, indicating that detached petal discs are still very sensitive to *B. cinerea* and that the defense signaling molecule JA is further induced upon infection beyond the levels caused by the process of obtaining discs.

### TRV-based VIGS for the rapid and high-throughput screening of rose genes in *B. cinerea* resistance

VIGS is a rapid and powerful tool for reverse genetics in various plant species, especially those in which genetic transformation is inconvenient or laborious. In rose, we previously devised a grafting VIGS method in which axillary sprouts were cut off and vacuum infiltrated. The inoculated scions were then grafted back onto the plants for flowering. Flower silencing phenotypes could be observed within four or five weeks of infiltration. We have shown that this grafting VIGS method can be used to efficiently silence genes related to floral color, development, and fragrance, providing a relatively fast method for assessing the functions of genes in roses^12^.

Grafting VIGS in intact rose flowers can certainly also be used to study *B. cinerea* resistance. However, the establishment of VIGS in intact rose flowers takes several weeks (until the agroinoculated sprout flower) and requires large-scale facilities for growing many plants and a great deal of work. By contrast, the silencing of candidate genes in petal discs can be accomplished in a matter of days and uses fewer resources. In the present study, by silencing *RhLOX5* and *EIN3*, which are genes required for JA- and ET-mediated resistance to *B. cinerea*, respectively, we demonstrated that the DPDA in combination with VIGS in petal discs can be used to elucidate defense signaling against *B. cinerea* in rose flowers. In addition, although the silencing of *RhDFR1* induces bleached petals in complete rose flowers^12^, this gene is not a suitable VIGS reporter for petal discs. However, we showed that the transient silencing of *RhPR10.1* promoted accelerated color-fading phenotypes of petal discs, suggesting that this gene can be used as a visible reporter for VIGS in petal discs.

TRV-derived VIGS has been successfully applied to many plant species. However, the high-throughput screening of gene function using VIGS remains challenging in many plant species owing to time, labor, and space requirements. A high-throughput screen of gene function by VIGS has been reported only in *N. benthamiana*, in which it was originally developed^20,21^. Considering that agroinoculated petal discs can be conveniently and efficiently silenced within several days and require relatively little space in a climate room, the VIGS and DPDA methods described herein can potentially be used not only for the functional analysis of a single gene but also for high-throughput screening of a large number of candidate genes involved in *B. cinerea* resistance. Indeed, in this study, we used the DPDA combined with VIGS to identify two novel transcription factor genes not previously known to be involved in *B. cinerea* resistance in rose.

## Materials and methods

### Plant materials

*Rosa hybrida* ‘Samantha’ plants were grown in soil in the greenhouse under the following conditions: 20 to 25 °C with 50% to 70% relative humidity and 16 h/8 h day/night periods. Cut roses were harvested from the greenhouse at stage 2 of flower opening and immediately placed in water. All other rose cultivars were ordered from the Kunming International Flora Auction Trading Center and delivered to the lab within 24 h. The flower stems were recut to 20 cm in length under water and placed in deionized water. After treatment, the flowers were placed in a vase with deionized water under controlled conditions of 23–25 °C with 30–40% relative humidity and 12 h/12 h day/night periods to await further processing.

### *B. cinerea* inoculation

*B. cinerea* strain B05.10 or CAU8324 was grown on potato dextrose agar at 22 °C*. B. cinerea* conidia were harvested from 10- to 14-day-old fungal plates and washed with tap water. The conidia were suspended at the desired final concentration in half-strength potato dextrose broth (PDB). For inoculation, rose petals were punched into 16 mm discs and rinsed with deionized water. Then, 2 μL of a conidial suspension was dropped onto the adaxial surface of the punched petals. As a control, petals were mock-inoculated with half-strength PDB. After (mock) inoculation, the petals were transferred to agar plates. The inoculated petals were evaluated by observing disease symptoms at 60 hpi. Each inoculation was repeated at least three times using a total of at least 48 discs. Student’s *t* test was used for statistical analysis. For the measurement of ion leakage, petal discs from each treatment were placed in 10 mL of 0.4 m mannitol and shaken at 40 rpm for 3 h. A conductivity meter (DDBJ-350; LeiCi) was used to measure the conductivity of the solution.

### Detection of phytohormones in rose petals

The plant samples were ground to a powder in liquid nitrogen. Then, 500 μL of extraction buffer (2-propanol/H_2_O/HCl [2:1:0.002, v/v/v]) was added, and the samples were vortexed for 10 s and shaken for 30 min at 4 °C. Next, 1 mL of CHCl_3_ was added, and the solution was vortexed for 10 s, shaken for 30 min at 4 °C, and then centrifuged at 14,000 rpm for 5 min at 4 °C. Finally, 1.2 mL of the lower layer of the liquid was transferred to a fresh test tube and dried at room temperature under nitrogen. The dried sample was resuspended in 0.1 mL of methanol and assayed by liquid chromatography-mass spectrometry (HPLC Shim-pack UFLC SHIMADZU CBM30A system; MS, Applied Biosystems 6500 Triple Quadrupole).

### Hormone treatments of rose flowers

For the hormone treatments, flower stems were placed in vases containing aqueous solutions of JA (50 µM), salicylic acid (50 and 100 µM), or 1-aminocyclopropane-1-carboxylic acid (ACC; 50, 100, 20, and 400 µM), or in deionized water as a control, for 24 h. The flower petals were then punched into discs for inoculation.

### Ethylene and 1-MCP treatment of rose flowers

Ethylene and 1-MCP were applied to the roses as described previously^13^. Briefly, rose flowers were exposed to ethylene (10 μL/L), 1-MCP (2 μL/L), or regular air as a control for 24 h. A 1 M NaOH solution was added to the chambers to prevent CO_2_ accumulation. The flower petals were then punched into discs for inoculation.

### VIGS

To obtain the *TRV::RhLOX5* construct, a 461-bp fragment from the 3’ UTR of *RhLOX5* was cloned into the TRV vector *pTRV2* and then electroporated into *Agrobacterium* strain GV3011. The TRV constructs *TRV::00*, *TRV::RhPR10.1*, and *TRV::RhEIN3* have been described previously^15,16,22^. To establish VIGS in rose petals, detached petals were obtained from the outermost whorls of rose flowers at stage 2 of flower opening. Then, a 15-mm disc was punched from the center of each petal. The petal discs were vacuum infiltrated with *Agrobacterium* carrying TRV constructs as described by Zhang and Thomma^14^. Briefly, an overnight culture of *Agrobacterium* was harvested at an OD_600_ of 0.8 by centrifugation at 4000 rpm for 1 min and then resuspended in the infiltration buffer (IB; 20 g sucrose, 5 g Murashige and Skoog medium without vitamins, 10 mL of 1 M *N*-morpholinoethanesulfonic acid, 1 mL of 0.2 M acetosyringone) to a final OD_600_ of 2. *Agrobacterium* cultures containing constructs expressing *TRV1* and *TRV2* were mixed in a 1:1 ratio and vacuum infiltrated into petal discs. The petal discs were immersed in the bacterial suspension, and infiltration was performed under vacuum at 0.7 MPa. After the release of the vacuum, infiltrated petal discs were washed with deionized water. After TRV infection, the petal discs were left under dark conditions at 8 °C for 3 days. Then, the discs were placed in a climate chamber at 22 °C/19 °C with 16 h/8 h day/night periods for 3 days. At 6 days post-TRV infection, the petal discs were inoculated with *B. cinerea*. VIGS was repeated at least three times using at least 16 discs in each experiment. After *B. cinerea* inoculation, Student’s *t*-test was conducted. All primers are listed in Supplemental Table S1.

### RNA extraction and qPCR

Total RNA was extracted from rose petal discs as described previously^13^. First-strand cDNA was synthesized from 1 μg of total RNA using the Takara Reverse Transcriptase M-MLV cDNA Synthesis Kit (Takara, Dalian, China) according to the manufacturer’s instructions. One microliter of first-strand cDNA was used as a template in the reaction with the KAPA SYBR Quantitative PCR Kit (Kapa Biosystems), which was run in a StepOnePlus Real-Time PCR System (Thermo Fisher Scientific). *RhACT5* was used as an internal housekeeping gene control as described previously^12^. All primers used for qPCR are listed in Supplemental Table S1.

## Supplementary information

Supplemental Figure S1

Supplemental Figure S2

Supplemental Figure S3

Supplemental Figure S4

Supplemental Figure S5

Supplementary Table S1. Primers used in this study

Supplementary Table S2. Accession numbers of the sequences included in this study
