# Supplementary figures and images for: A detached petal disc assay and virus-induced gene silencing facilitate the study of Botrytis cinerea resistance in rose flowers
Source: Hortic Res. 2019 Dec 1;6:136. doi: 10.1038/s41438-019-0219-2 (PMC6885046; doi:10.1038/s41438-019-0219-2)

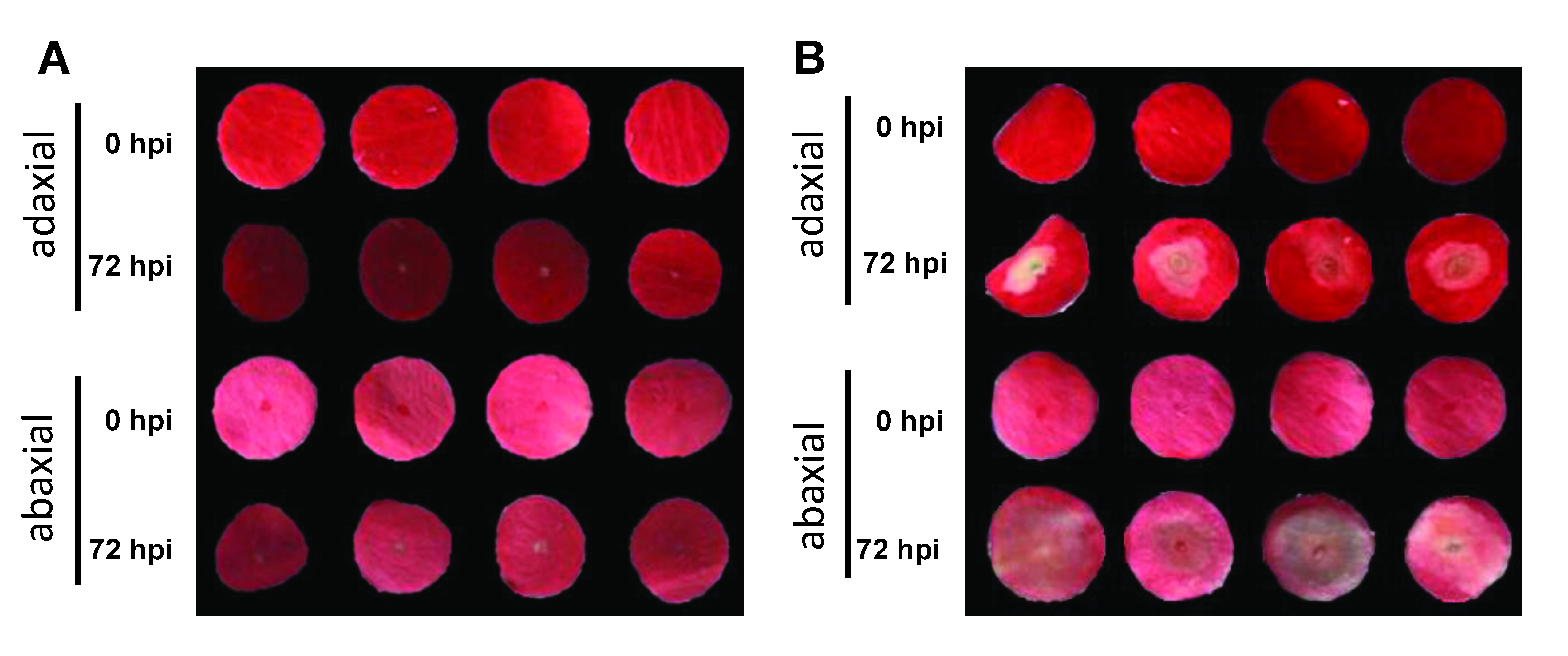

Supplement: Supplementary file 1 — Supplemental Figure S1 [file 41438_2019_219_MOESM1_ESM.tif]

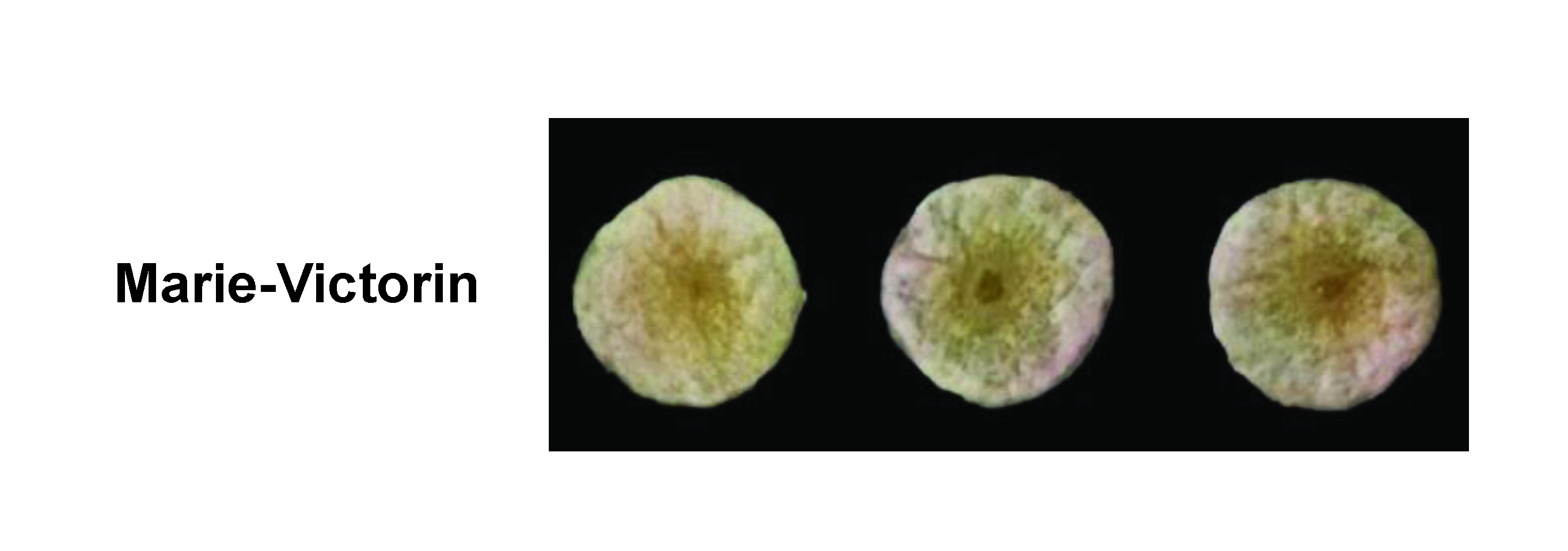

Supplement: Supplementary file 2 — Supplemental Figure S2 [file 41438_2019_219_MOESM2_ESM.tif]

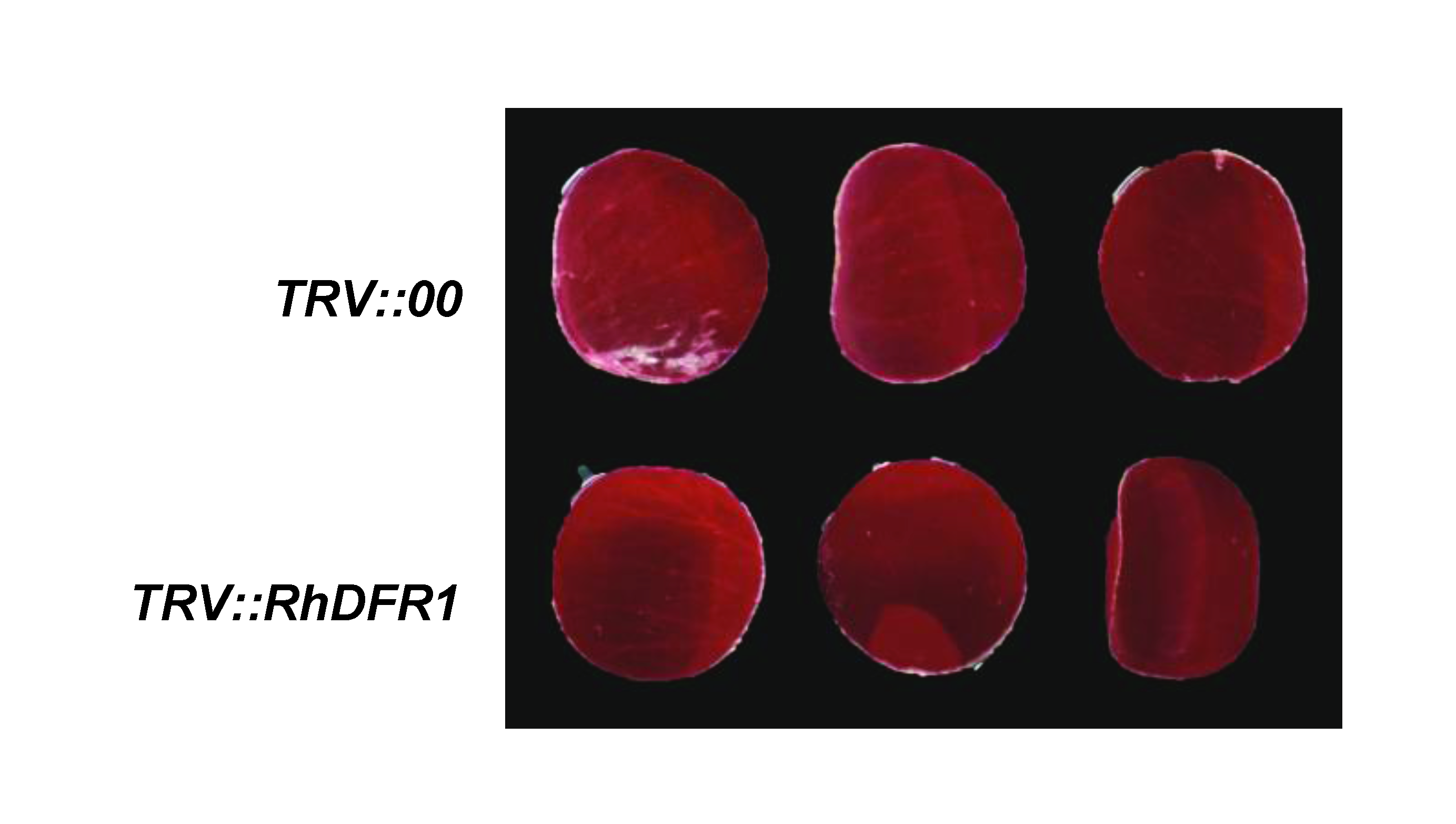

Supplement: Supplementary file 3 — Supplemental Figure S3 [file 41438_2019_219_MOESM3_ESM.tif]

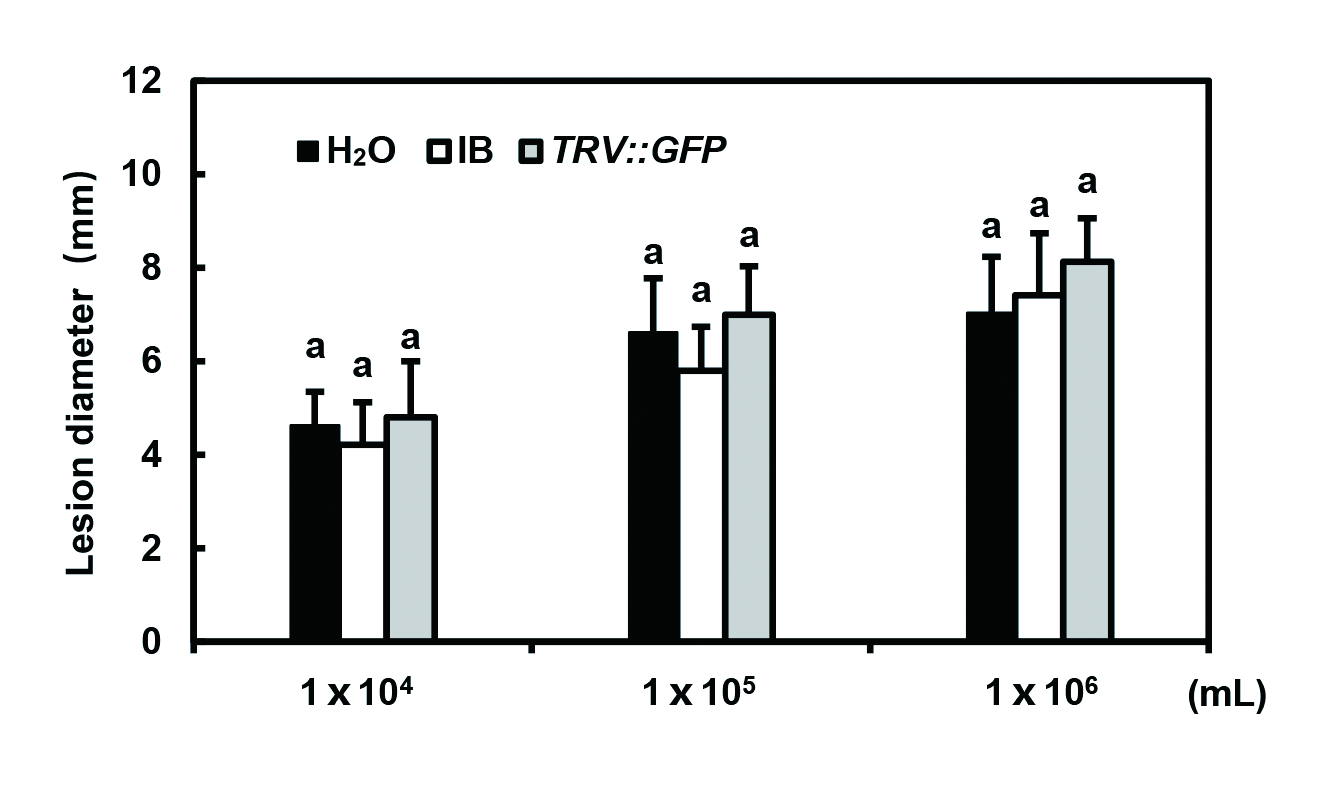

Supplement: Supplementary file 4 — Supplemental Figure S4 [file 41438_2019_219_MOESM4_ESM.tif]

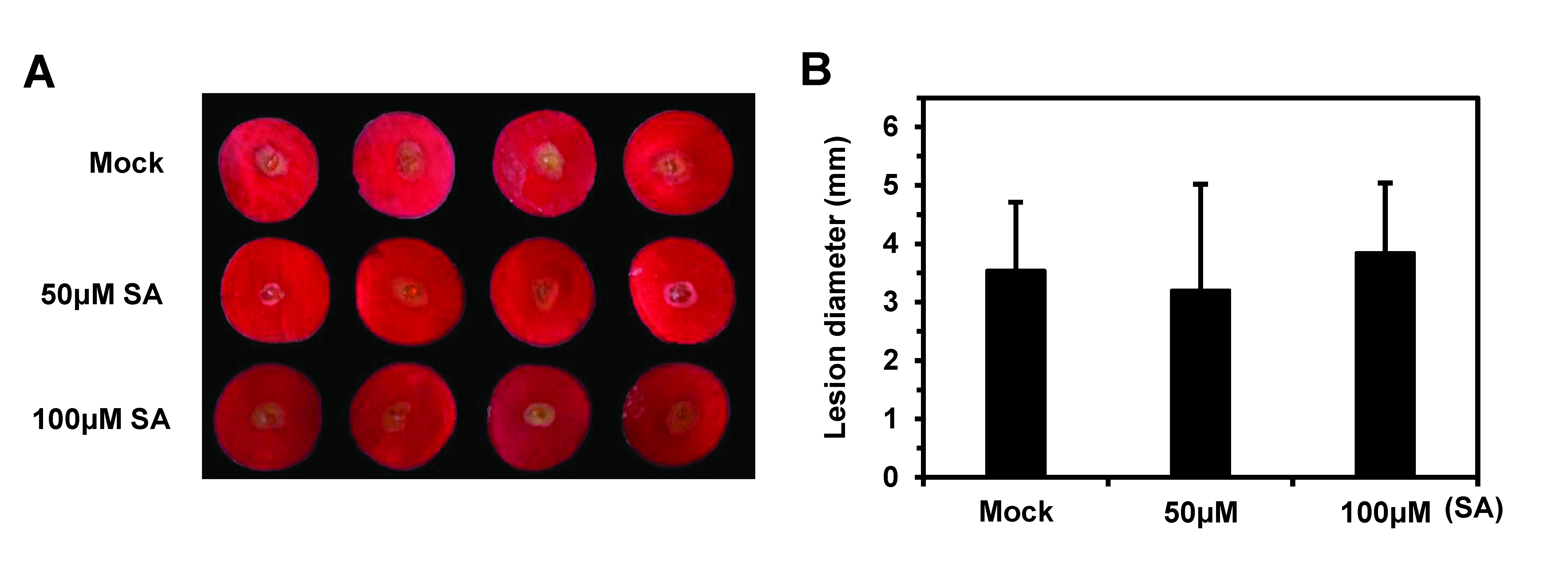

Supplement: Supplementary file 5 — Supplemental Figure S5 [file 41438_2019_219_MOESM5_ESM.tif]
