# Supplementary material for: A detached petal disc assay and virus-induced gene silencing facilitate the study of Botrytis cinerea resistance in rose flowers
Source: Hortic Res. 2019 Dec 1;6:136. doi: 10.1038/s41438-019-0219-2 (PMC6885046; doi:10.1038/s41438-019-0219-2)
Supplement: Supplementary file 6 — Supplementary Table S1. Primers used in this study [file 41438_2019_219_MOESM6_ESM.docx]

**Supplementary Table S1.** Primers used in this study

| **Primer name** | **Sequence (5’-3’)** |
| --- | --- |
| attB1-TRV-RhLOX5 | GGGGACAAGTTTGTACAAAAAAGCAGGCTCCCAGGCACTGCTGAATATAATGAAC |
| attB2-TRV-RhLOX5 | GGGGACCACTTTGTACAAGAAAGCTGGGTCGGGATAGAGCAAGGTATAAGGC |
| attB1-TRV-RhERF096 | GGGGACAAGTTTGTACAAAAAAGCAGGCTAGCAGCTAGAGCTTATGACA |
| attB2-TRV-RhERF096 | GGGGACCACTTTGTACAAGAAAGCTGGGTATCTTCACAATCAAGCAGTT |
| attB1-TRV-RhERF027 | GGGGACAAGTTTGTACAAAAAAGCAGGCTAGGAAAATGGGTGTCGGAAA |
| attB2-TRV-RhERF027 | GGGGACCACTTTGTACAAGAAAGCTGGGTTTGTCCAACATTTGAAGTCT |
| attB1-TRV-RhWRKY36 | GGGGACAAGTTTGTACAAAAAAGCAGGCTGAACCATCCGATACAACAAG |
| attB2-TRV-RhWRKY36 | GGGGACCACTTTGTACAAGAAAGCTGGGTTTTGTCAAGTCAAGAGTGAT |
| attB1-TRV-RhTGA2 | GGGGACAAGTTTGTACAAAAAAGCAGGCTAGGCCTTGCAACAATCTCTA |
| attB2-TRV-RhTGA2 | GGGGACCACTTTGTACAAGAAAGCTGGGTCAAAAGCGTTTTATTCTCTT |
| attB1-TRV-RhMYB44 | GGGGACAAGTTTGTACAAAAAAGCAGGCTCTCTGCCTCAACATGTACTA |
| attB2-TRV-RhMYB44 | GGGGACCACTTTGTACAAGAAAGCTGGGTATTTTGATCTTCTCCAGACA |
| attB1-TRV-RhWRKY75 | GGGGACAAGTTTGTACAAAAAAGCAGGCTGCACCAAGCTAGCAACAATA |
| attB2-TRV-RhWRKY75 | GGGGACCACTTTGTACAAGAAAGCTGGGTTTCATATGTAGTCACCACAA |
| attB1-TRV-RhHFA4B | GGGGACAAGTTTGTACAAAAAAGCAGGCTGGCTTGAGAAAATGGAGTCT |
| attB2-TRV-RhHFA4B | GGGGACCACTTTGTACAAGAAAGCTGGGTATCAAGTCCTGAAGATTTAG |
| attB1-TRV-RhbHLH92 | GGGGACAAGTTTGTACAAAAAAGCAGGCTGTGATAAGAATTGGATTGTG |
| attB2-TRV-RhbHLH92 | GGGGACCACTTTGTACAAGAAAGCTGGGTATTCAATTTCCACTTCTGCA |
| attB1-TRV-RhMYB4 | GGGGACAAGTTTGTACAAAAAAGCAGGCTTGGATGAATTACTTGAGGCC |
| attB2-TRV-RhMYB4 | GGGGACCACTTTGTACAAGAAAGCTGGGTTGATGAAGAGAAGTTTTCTC |
| attB1-TRV-RhTGT3B | GGGGACAAGTTTGTACAAAAAAGCAGGCTAAACATTGGAAGCAGCACTG |
| attB2-TRV-RhTGT3B | GGGGACCACTTTGTACAAGAAAGCTGGGTTAGCTAATTGATGAGAACAA |
| RhActin5-F | GAGCGTTTCAGATGCCCAGA’ |
| RhActin5-R | TGGTGGGGCAACCACCTTA’ |
| RhPR10.1-F | AAGAGCCGCTGGTCTGTTC’ |
| RhPR10.1-R | GCAAGCCAAAACAATCTCCG’ |
| RhLOX5-F | GAAATGGTGGGAAGAGCTCCGCAAT’ |
| RhLOX5-R | GCCATTGCCCGAAATTCACAGCTGC’ |
| RhEIN3-F | TAAGGAGAGTTCTACCTGGCTGGCC’ |
| RhEIN3-R | CTCATCTTCAGCCCCTTCAACATCA |
