# Supplementary material for: A detached petal disc assay and virus-induced gene silencing facilitate the study of Botrytis cinerea resistance in rose flowers
Source: Hortic Res. 2019 Dec 1;6:136. doi: 10.1038/s41438-019-0219-2 (PMC6885046; doi:10.1038/s41438-019-0219-2)
Supplement: Supplementary file 7 — Supplementary Table S2. Accession numbers of the sequences included in this study [file 41438_2019_219_MOESM7_ESM.docx]

**Supplementary Table S2.** Accession numbers of the sequences included in this study

| **Gene name** | **Accession number** |
| --- | --- |
| RhERF096 | RchiOBHm_Chr4g0423581 |
| RhERF027 | RchiOBHm_Chr7g0199231 |
| RhWRKY36 | RchiOBHm_Chr2g0133001 |
| RhTGA2 | RchiOBHm_Chr1g0325831 |
| RhMYB44 | RchiOBHm_Chr7g0219951 |
| RhWRKY75 | RchiOBHm_Chr4g0429851 |
| RhHFA4B | RchiOBHm_Chr2g0131371 |
| RhbHLH92 | RchiOBHm_Chr4g0390311 |
| RhMYB4 | RchiOBHm_Chr6g0288151 |
| RhTGT3B | RchiOBHm_Chr3g0462911 |
| RhPR10.1 | RchiOBHm_Chr4g0423941 |
| RhLOX5 | RchiOBHm_Chr4g0416591 |
| RhEIN3 | RchiOBHm_Chr5g0049961 |
| RhActin5 | [RchiOBHm_Chr3g0466761](https://lipm-browsers.toulouse.inra.fr/pub/RchiOBHm-V2/jbrowse/current/?data=../data/RchiOBHm-V2&loc=RchiOBHmChr3:13130331..13140350&highlight=RchiOBHmChr3:13134568..13137575) |
